# Assessment and Quantification of Foam Cells and Lipid Droplet–Accumulating Microglia in Mouse Brain Tissue Using BODIPY Staining

**DOI:** 10.21769/BioProtoc.5107

**Published:** 2024-11-05

**Authors:** Boaz K. Maiyo, Sanna H. Loppi, Helena W. Morrison, Kristian P. Doyle

**Affiliations:** 1 Department of Immunobiology, University of Arizona, Tucson, AZ, USA; 2 College of Nursing, University of Arizona, Tucson, AZ, USA; 3 Department of Neurology, University of Arizona, Tucson, AZ, USA; 4 BIO5 Institute, University of Arizona, Tucson, AZ, USA; 5 Arizona Center on Aging, University of Arizona, Tucson, AZ, USA; 6 Department of Psychology, University of Arizona, Tucson, AZ, USA; 7 Department of Neurosurgery, University of Arizona, Tucson, AZ, USA

**Keywords:** Foam cells, Microglia, Macrophage, Myelin, Boron-dipyrromethene, BODIPY, CNS

## Abstract

This paper presents a refined, user-friendly protocol for using boron-dipyrromethene (BODIPY) to assess and quantify foam cells and lipid droplet–accumulating microglia (LDAM) in mouse brain tissue. The protocol aims to enhance existing methodologies by offering precise and efficient evaluation of foam cells and LDAM burden in various neuropathological conditions linked to lipid metabolism and neuroinflammation. A notable challenge in analyzing tissue from mouse models of these neurodegenerative disorders is the interference caused by the autofluorescent molecule lipofuscin. Our protocol addresses this issue with specific steps that effectively distinguish BODIPY fluorescence from lipofuscin autofluorescence, using advanced imaging techniques and filter settings to ensure accurate and reliable analysis. By providing a straightforward and accessible method, this research aims to facilitate the broader adoption of BODIPY-based techniques for detailed foam cell and LDAM analysis in mouse brain tissue, potentially enhancing diagnostic capabilities and deepening our understanding of how these cells contribute to neurodegenerative disease mechanisms.

Key features

• To induce foam cell/LDAM CNS formation, this protocol was developed using brain tissue from mice subjected to permanent occlusion of the middle cerebral artery.

• The protocol utilizes mouse brain tissue that is fixed in 4% PFA.

• Additional markers, CD68 and Iba1, are incorporated to evaluate myeloid cell lineage.

• The protocol includes a simple method for distinguishing BODIPY fluorescence from autofluorescence.

## Background

Foam cell accumulation, resulting from the overwhelmed processing of myelin-derived lipids by macrophages and microglia, plays a pivotal role in multiple pathological conditions affecting the central nervous system (CNS), including stroke, spinal cord injury, and multiple sclerosis (MS) [1–3]. In the aging brain, lipid droplet–accumulating microglia (LDAM) signify a dysfunctional and pro-inflammatory state [4]. However, myeloid cells that accumulate lipid droplets often exhibit autofluorescence due to the presence of lipofuscin, complicating accurate visualization and quantification. Currently, there is a lack of user-friendly assessment protocols for accurately measuring the burden of foam cells and LDAM in mouse models of neurological disorders. The goal of this paper is to bridge this gap by introducing a comprehensive and accessible boron-dipyrromethene (BODIPY)-based protocol tailored for the analysis of foam cells and LDAM in highly autofluorescent mouse brain tissue.

The BODIPY-based approach outlined in this study leverages the distinctive properties of BODIPY dyes, especially BODIPY 493/503, for specific neutral lipid staining and efficient visualization of foam cells. Particularly well-suited for fluorescence microscopy, this technique offers a level of detail unmatched by alternative methods like Oil Red O or H&E staining. The BODIPY-based staining method we describe is rapid, straightforward, and cost-effective. Additionally, BODIPY staining can be combined with immunostaining for a more comprehensive analysis of myeloid cell phenotype. However, despite the relatively narrow emission spectrum of BODIPY 493/503, peaking at around 503 nm, careful deployment is essential to mitigate interference from the autofluorescent molecule lipofuscin, which has a heterogeneous emission spectrum ranging from 480 to 695 nm [5]. This creates the potential for spectral overlap, even though the emission spectra do not perfectly align.

By presenting a protocol that overcomes the limitations posed by lipofuscin autofluorescence, this study aims to empower researchers with a robust and user-friendly fluorescent tool for studying foam cell and lipid droplet load in mouse brain tissue. The accessibility of this BODIPY-based protocol may encourage broader adoption and implementation, thereby promoting advancements in our understanding of the relationship between foam cells and LDAM accumulation in the aging CNS and various neurological disorders.

## Materials and reagents

**Biological materials**

1. Tissue sections: 40 µm thick sections from saline perfused C57BL/6 mice (Jackson Laboratories, catalog number: 00064), fixed in 4% paraformaldehyde for 24 h and cryopreserved in 30% sucrose using standard techniques. These mice were sacrificed seven weeks post a distal middle cerebral artery occlusion model of stroke, as described by Doyle et al. [6]

**Reagents**

1. Rat anti-mouse CD68 (Bio-Rad, catalog number: MCA1957GA)
2. Rabbit anti-mouse Iba1 (FUJIFILM Wako Pure Chemical Corporation, catalog number: 019-19741)
3. Goat anti-rabbit IgG (H+L) cross-adsorbed secondary antibody, Alexa Fluor^TM^ 568 (Thermo Fisher Scientific, catalog number: A-11011)
4. Goat anti-rat IgG (H+L) cross-adsorbed secondary antibody, Alexa Fluor^TM^ 647 (Thermo Fisher Scientific, catalog number: A-21247)
5. BODIPY 493/503 (4,4-Difluoro-1,3,5,7,8-Pentamethyl-4-Bora-3a,4a-Diaza-*s*-Indacene) (Thermo Fisher Scientific, catalog number: D3922)
6. Dimethyl sulfoxide (DMSO) (Thermo Fisher Scientific, Gibco^®^, catalog number:25200-056)
7. Antifade mounting medium (Vector Laboratories, SKU: H-1400-10)
8. 10× PBS buffer pH 7.4 (Thermo Fisher Scientific, catalog number: AM9625)
9. Goat serum (Atlanta Biologicals, catalog number: S131x)
10. Triton X-100 (MilliporeSigma, catalog number: 9002-93-1)

**Solutions**

1. 0.1 M PBS (see Recipes)
2. 0.3% Triton X-100 in 0.1 M PBS (see Recipes)

**Recipes**

1. **0.1 M PBS**
  **Table:** 

Reagent	Final concentration	Amount
10× PBS	10%	100 mL
Double-distilled H_2_O	90%	900 mL
Total	10%	1,000 mL
2. **0.3% Triton X-100 in 0.1 M PBS**
  **Table:** 

Reagent	Final concentration	Amount
0.1 M PBS	99.7%	498.5 mL
Triton X-100	0.3%	1.5 mL
Total	0.3%	1,000 mL

**Laboratory supplies**

1. 12-well tissue culture plate (CELLTREAT Scientific Products, catalog number: 229112)
2. Brushes for tissue handling (Precisionary, SKU: VF-VM-PB-CANAL)
3. Hydrophobic barrier pap pen (Thermo Fisher Scientific, catalog number: R3777)
4. Round cover glass, #1.5 thickness, 12 mm, 100 pack (Fisher Scientific, Thomas Scientific, catalog number: NC1129240)
5. Superfrost Plus^TM^ microscope slides white tab (Fisher Scientific, Fisherbrand, catalog number: 1255015)
6. Micro-centrifuge transfer pipette (RPI Research Products International, SKU: 147500)
7. 15 mL conical centrifuge tubes (Fisher Scientific, Falcon^TM^, catalog number: 14-959-53A)
8. 10 μL micropipette tips (USA Scientific, catalog number: 1161-3700)
9. 200 μL micropipette tips (USA Scientific, catalog number: 1163-1700)
10. 1,250 μL micropipette tips (USA Scientific, catalog number: 1161-1820)

## Equipment

1. Digital orbital shaker (Labnique^TM^, catalog number: MT-201-BX)
2. Leica DM6000B microscope (Leica Microsystems, SKU: 14628696)
3. Zeiss LSM880 NLO upright multiphoton/confocal microscope

## Software and datasets

1. ImageJ (free, https://imagej.net/software/fiji/downloads, Fiji version, 2024)

## Procedure

1. Select infarcted mouse brain tissue sections.
*Note: If only staining for BODIPY, skip to step 8.*
2. Place tissue in a 12-well tissue culture plate and wash three times for 10 min each in 0.1 M PBS at room temperature (RT) on a shaker.
3. Blocking: Block for 1 h at RT with 10% goat serum and 0.3% Triton X-100 in 0.1 M PBS.
*Tip: Make sure the blocking solution uses serum from the species in which the secondary antibody was generated.*
4. Primary antibody: Incubate overnight at room temperature on a rotator in 3% goat serum and 0.3% Triton X-100 in 0.1 M PBS containing: 1:500 anti-CD68 made in rat and 1:500 anti-Iba1 made in rabbit.
*Tip: If the staining is faint, increase the antibody concentration. If there is excessive background, reduce the antibody concentration. The recommended dilution is 1:500–1,000. Another alternative when staining is too faint is to increase the incubation time to 48–72 h.*
5. Wash three times for 10 min each in 0.1 M PBS at RT on a shaker.
6. Secondary antibody: Incubate for 2 h at RT in 3% goat serum + 0.3% Triton X-100 in 0.1 M PBS with 1:500 goat anti-rabbit and 1:500 goat anti-rat.
*Tip: If the staining is faint, increase the antibody concentration. If there is excessive background, reduce the antibody concentration. The recommended dilution is 1:500–1,000. Another alternative is to increase the incubation time to 4 h.*
*Note: From this point onward, keep samples protected from light.*
7. Wash three times for 10 min each in 0.1 M PBS at RT on a shaker.
8. Mounting: Gently place tissue sections in 0.1 M PBS and mount them on slides.
*Notes:*
  - *Air dry slides for approximately 30 min.*
  - *To avoid drying the tissue, do not leave the slides out for more than 1 h.*
9. Draw a hydrophobic barrier around the sections with the hydrophobic pen.
10. First washing: Wash the sections three times for 10 min each in 0.1 M PBS at RT.
11. Staining: Incubate the sections in a solution of 1:25 BODIPY 492/515 in DMSO for 30 min, protected from light.
12. Second washing: Wash the sections three times for 10 min each in 0.1 M PBS at RT, protected from light.
13. Cover slipping:
  - Mount the sections with antifade mounting media.
  - Incubate for 1–2 h at RT, protected from light.
14. Imaging: Detect the fluorescence emission of BODIPY 493/503-stained samples using a fluorescence microscope equipped with a 493 nm excitation filter and a 503 nm emission filter.

## Data analysis

This section outlines the analysis of cellular lipids and lipid droplets stained with BODIPY 493/503 within a specified area using the ImageJ software, a method previously applied by Loppi et al. [7].

1. Open the program.
2. Click *File*, then *Open*, and select the desired image.
3. The image appears on the screen.
4. To select the desired area, click on the *Polygon selections* on the toolbar and manually outline the area.
  • It should appear as a polygon icon with defined sides.
*Note: If there is no need to select a smaller area of the image, this step can be omitted.*
5. Select *Image, Type*, and *8-bit*.
  • The image should now become black and white.
6. Continue by clicking *Image, Adjust*, and *Threshold*.
7. A screen will appear that will allow you to select positive staining.
  • Adjust the Min and Max threshold values to a level where only the stained areas are colored.
*Tip: Set the Max to 255 and slowly lower the min for finer selections.*
8. To quantify the selection, click *Analyze* and *Measure*.
  • A screen will appear with the name of the image, as well as the Area, Mean, Min, Max, %Area, MinThr, and MaxThr.
*Tip: The desired measurements can be selected by clicking* Analyze *and* Set Measurements.
9. Copy the data to an Excel spreadsheet.
10. Close the image.
11. Repeat steps 2–10 until all images are analyzed.
*Tip: For accurate comparison between images, keep the Min and Max threshold values the same between images.*
12. Use the *%Area* value for comparisons.
13. Calculate the average %Area for each group of brain sections (e.g., control vs. treated).
14. Perform statistical analysis to determine whether there is a significant difference in lipid accumulation between groups.

## Validation of protocol

**Autofluorescence control**

**Figure 1.**
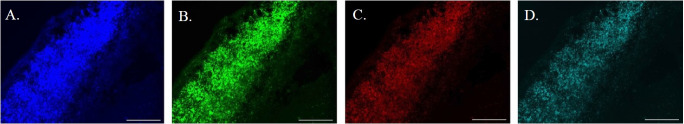
The broad spectrum of lipofuscin autofluorescence. A. Lipofuscin-rich infarcted region of a brain section from a stroked mouse, imaged with an exposure time of 1.2 s, excitation of 355–425 nm, and emission of 470 nm. B. The same section imaged with an exposure time of 1.2 s, excitation of 460–500 nm, and emission of 512–542 nm. C. The same section imaged with an increased exposure time of 3 s, excitation of 540–580 nm, and emission of 590–650 nm. D. The same section imaged with an increased exposure time of 10 s, excitation of 590–650 nm, and emission of 662–738 nm. All images were captured at 10× magnification with the scale bar representing 300 µm.

**Background:** Autofluorescence, predominantly from lipofuscin, presents a significant challenge for researchers studying injured and diseased brain tissue, as it occurs across a spectrum from 480 to 695 nm [5]. It can be observed by imaging an unstained section through multiple filter cubes with different excitation and emission settings. Lipofuscin autofluorescence is most intense at lower wavelengths and gradually diminishes as wavelengths increase, necessitating longer exposure times for accurate capture (Figure 1A–D). Considering that the BODIPY 493/503 stain emits at 503 nm, it is crucial to ensure that the stained areas accurately represent lipids in the brain rather than artifacts from lipofuscin autofluorescence.

**Experimental setup:** To address the issue of autofluorescence, we conducted a control experiment using two brain tissue samples from the same experimental group. One sample was stained with BODIPY according to the described protocol while the other was only incubated in PBS for the same duration instead of the BODIPY solution. Both samples were imaged using a Leica microscope the following day.

**Figure 2.**
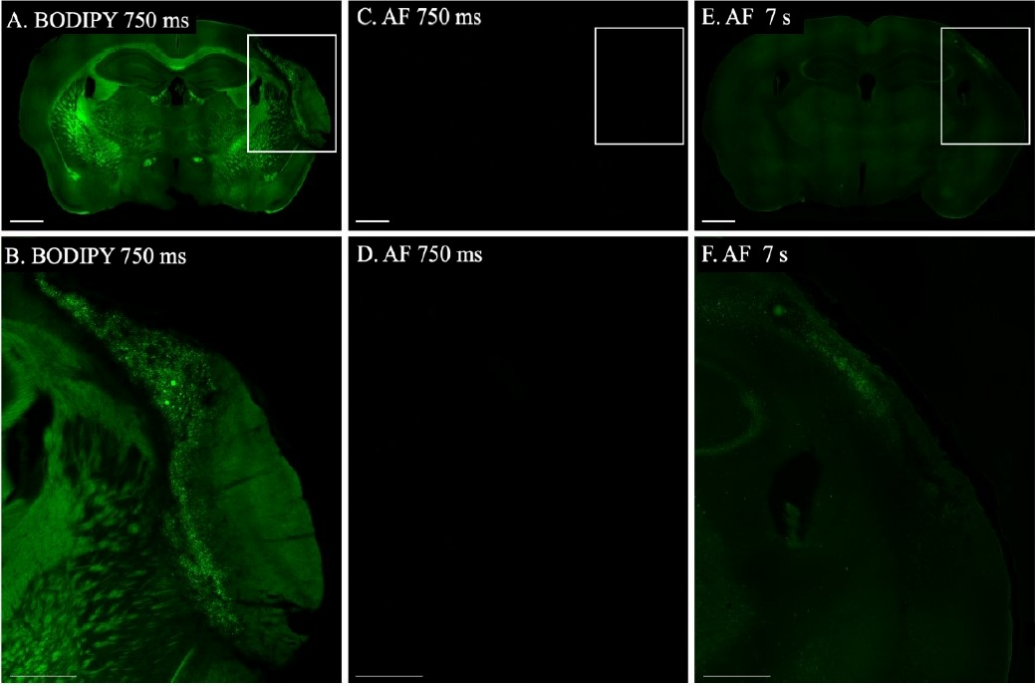
Autofluorescence control. A. Whole-brain section stained with BODIPY, imaged at 10× magnification with an exposure time of 750 ms and a gain of 1.5. B. Enlarged view of the infarct from image A, using the same imaging parameters. C. Section processed with the BODIPY protocol but not stained with BODIPY, imaged at 10× magnification with an exposure time of 750 ms and a gain of 1.5. D. Enlarged view of the infarct area from image C. E. The same unstained section as in C, imaged at 10× magnification with an exposure time of 7 s and a gain of 1.5 to reveal autofluorescence. F. Enlarged view of the infarct area from image E. Scale bars: 1 mm for images A, C, and E; 500 μm for images B, D, and F.

**Imaging protocol:** The BODIPY-stained section was initially imaged at 10× magnification with an exposure time of 750 ms and a gain of 1.5, which provided clear visualization of the stain (Figure 2A). We then increased the magnification to 20×, achieving an even clearer image with an exposure time of 576.07 ms and the same gain (Figure 2B). In contrast, the control section, imaged under identical conditions at both 10× and 20× magnifications, showed no discernible signal (Figure 2C and D). Only when the exposure time was extended to 7 s at 10× and 5 s at 20× did lipofuscin autofluorescence become visible (Figure 2E and F), thereby confirming the specificity of the BODIPY staining over lipofuscin autofluorescence.

**Lipid droplet visualization within myeloid cells**

When microglia and macrophages are overwhelmed by myelin debris following a stroke, they transform into foam cells. This transformation not only results in a loss of immune function but also activates pathways that may contribute to tissue damage [2]. To validate the use of the BODIPY protocol for visualizing lipid droplets within myeloid cells, stroked brain tissue was immunostained with antibodies specific for IBA1 and CD68 by using standard techniques described by Potts et al. [8]. IBA is a pan surface marker for microglia, while CD68 serves as an intracellular marker predominantly found in the lysosomes of monocytes and macrophages, commonly used to identify activated, phagocytic microglia and macrophages [9,10]. The tissue was subsequently stained with BODIPY and imaged.

**Figure 3.**
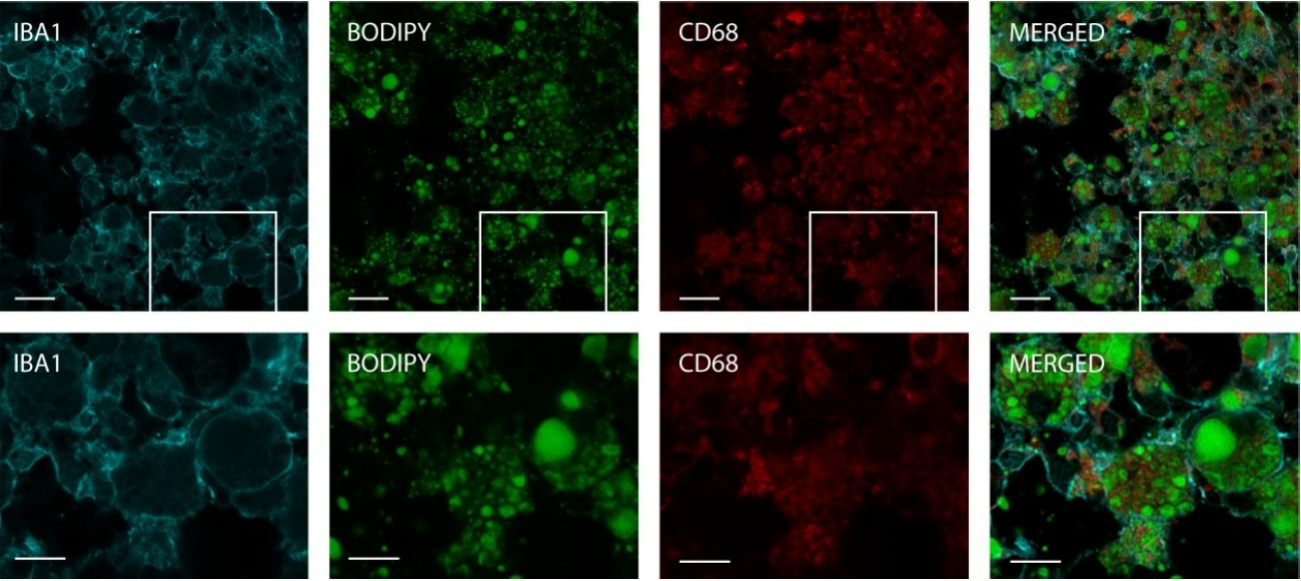
Intracellular staining of lipids in myeloid cells. The section depicted was stained with IBA1 (cyan), BODIPY (green), and CD68 (red), and captured using a Zeiss LSM880 NLO upright multiphoton/confocal microscope at 40× magnification with a 2× zoom. The top row images feature a scale bar of 20 μm, and the bottom row images feature a scale bar of 10 μm.

Imaging of the infarct revealed successful detection and visualization of BODIPY within IBA1 and CD68 positive cells. The IBA1 stain sharply outlined the myeloid cells, as depicted in Figure 3. BODIPY staining indicated a significant presence of lipid droplets within these cells. CD68 was also observed colocalized with the BODIPY stain within the myeloid cells, confirming the presence of BODIPY within the endosomal-lysosomal system of these cells. These findings validate the effectiveness of BODIPY staining for visualizing lipid-laden myeloid cells, including foam cells and LDAMs, in the context of neurological injury.

## General notes and troubleshooting

**Handling fluorescent stains and imaging guidelines**

Fluorescent stains gradually lose intensity over time; therefore, it is essential to image all sections within a set on the same day. Be aware that exposure times may vary slightly from day to day. To prevent photobleaching, avoid leaving sections under the microscope for extended periods, as prolonged exposure can cause tissue bleaching, evident as dark spots in the sections. Optimize your imaging process by starting with a lower exposure time to locate the desired area, then increase the exposure time only when ready to capture the image. If possible, perform imaging in a dark room to enhance the quality of your results. Always store slides at room temperature in a dark place to minimize light exposure.

**Co-staining protocol**

When performing co-staining with other markers using immunohistochemistry (IHC), it is advisable to complete the IHC first, followed by BODIPY staining. For colocalization, higher magnifications provide more precise images. Using 10× or 20× magnification may not yield satisfactory results. Instead, opt for 40× magnification and consider utilizing a Z-stack to achieve optimal colocalization accuracy.

**Troubleshooting**

**Issue:** Tissue clumping with the combined IHC and BODIPY staining protocol.

**Solution:** Pre-mounting instead of free floating.

1. Complete all IHC steps on pre-mounted slides and, following the washing steps, continue on to BODIPY staining.
2. Avoid using free-floating methods for IHC followed by BODIPY to avoid tissue clumping.
3. Rinse all tissue well to remove excess BODIPY staining.

**Issue:** The scale is unrecognized in the images.

**Solution:** Manually set the scale in ImageJ by using scale bar images for the magnification used. Follow these steps:

1. Open the scale bar images in ImageJ.
2. Use the *straight line tool* from the toolbar to draw a line over the scale bar.
3. Go to *Analyze* and select *Set Scale*.
  • A dialog box will appear.
4. In the dialog box, change the *Known distance* to the actual distance of the scale bar.
5. Set the *Unit of length* to µm and check the *Global* box.
  • Click OK.
  • The dialog box will automatically close.
6. Keep the scale bar image open in the background for the duration of the analysis. It must remain open as long as ImageJ is running.
*Note: Once set, the scale will apply to all images as long as ImageJ remains open, eliminating the need to reset the scale for each new image.*
